# Sex Cord-Stromal Tumors of Testis: A Clinicopathologic and Follow-Up Study of 15 Cases in a High-Volume Institute of China

**DOI:** 10.3389/fmed.2022.816012

**Published:** 2022-05-31

**Authors:** Yin Huang, Bo Chen, Dehong Cao, Zeyu Chen, Jin Li, Jianbing Guo, Qiang Dong, Qiang Wei, Liangren Liu

**Affiliations:** ^1^Department of Urology, West China Hospital, Institute of Urology, Sichuan University, Chengdu, China; ^2^West China School of Medicine, Sichuan University, Chengdu, China

**Keywords:** testis, testicular neoplasms, sex cord-stromal tumors, follow-up, orchiectomy

## Abstract

**Objectives:**

To report the first series of testicular sex cord-stromal tumors (TSCSTs) with detailed clinicopathologic findings and long-term follow-up in the Chinese population.

**Patients and Methods:**

From 2008 to 2018, 15 patients with TSCST were included in our study. The tumors were analyzed for epidemiological parameters, clinical characteristics, tumor markers, therapy, and follow-up data.

**Results:**

The median age of the patients was 28 years (range, 13–80 years). Para-aortic lymph node metastases were detected in 2 patients after radiological evaluation. Orchiectomy was performed in all patients, and the median diameter of the tumor was 1.5 cm (range, 0.5–5.0 cm). Nine Leydig cell tumors (LCTs), 5 Sertoli cell tumors (SCTs), and 1 unclassified type were confirmed after pathologic evaluation. Thirteen patients (86.7%) were categorized as stage I, and 2 patients (13.3%) were categorized as stage II. The median clinical follow-up was 39.0 months (range, 5–97 months), which showed 10 alive patients, such as 1 patient with progression at 40 months after orchiectomy. The 3- and 5-year progression-free survivals were 100 and 90.0%, respectively.

**Conclusion:**

Testicular sex cord-stromal tumor at stages I and II is a rare subtype with benign behavior and a favorable prognosis in the Chinese population. However, lymph node metastases may be the dominant risk factor for patients with TSCST.

## Introduction

Testicular sex cord-stromal tumor (TSCST) is a rare primary testicular neoplasm, accounting for approximately 4% of testicular tumors, while the remaining tumor is of germ cell origin. The most common subtype is Leydig cell tumor (LCT), accounting for 75% of TSCSTs, followed by Sertoli cell tumor (SCT), granulosa cell tumor (GCT), unclassified tumors, and mixed cell types (1, 2).

To date, TSCSTs have been poorly studied, and only a few case reports and small sample clinicopathologic studies have been published (3–13). Although there is a relatively large cross-sectional study of prognosis and cancer incidence based on the national cancer registry, the annual report from the National Central Cancer Registry of China unfortunately has limited information about testicular cancer (14). In this study, we aimed to present the clinicopathologic characteristics and survival of TSCSTs in the Chinese population.

In our study, we presented a series of 15 patients with TSCSTs from 2008 to 2018. All patients were registered in the Department of Urology, West China Hospital, Sichuan University. We reported the clinical and pathological findings, treatment, and prognostic outcomes observed in patients with TSCSTs, with the purpose of evaluating the efficacy of our clinical approach and contributing to the literature data.

## Materials and Methods

### Setting and Study Design

From January 2008 to December 2018, patients diagnosed with TSCST after surgical procedures at the Department of Urology, West China Hospital were included in our retrospective observational study. All clinical features (symptoms, physical signs, past medical history, and comorbidities), auxiliary examination results (hemogram, blood biochemistry, hormone assay, tumor markers [α-fetoprotein (AFP), β-human chorionic gonadotropin (β-HCG), and lactate dehydrogenase (LDH)], ultrasonography and radiology [computed tomography (CT) scan and magnetic resonance imaging (MRI)]), treatment (surgery and adjuvant therapy), and histopathological findings were retrieved from medical records if available. All diagnoses of TSCSTs were confirmed through postoperative pathological examination.

### Treatment and Follow-Up

Orchiectomy was the first-choice treatment for all patients enrolled in our study. For patients with a retroperitoneal disease on preoperative radiological evaluation, we recommended retroperitoneal lymph node dissection (RPLND) after orchiectomy. Tumors were staged according to the National Comprehensive Cancer Network staging system for testicular tumors (15). The classification of testicular tumors was confirmed through the WHO classification of testicular tumors (1).

Based on the studies of Silberstein et al. (4) and Kim et al. (9), patients were stratified into 2 groups by 6 high-risk features, such as (1) tumor greater than 5 cm, (2) necrosis, (3) moderate or severe nuclear atypia, (4) angiolymphatic invasion, (5) infiltrating margins, and (6) more than 5 mitotic features per 10 high-power fields. Patients with 0 or 1 high-risk features and no evidence of retroperitoneal disease on radiological evaluation were classified as the low-risk group, and patients with 2 or more high-risk features or retroperitoneal disease on radiological evaluation were classified as the high-risk group.

After completion of the therapy, patients were followed up regularly for symptoms, physical examination, scrotal ultrasound, and radiology. Total follow-up was calculated as the time from orchiectomy to the last follow-up. Progression was noted as an event and defined as emerging or recurrent disease-related symptoms or any change in tumors on radiology after diagnosis.

### Statistical Analysis

Progression-free survival (PFS) was calculated from the date of diagnosis to the date of progression and was estimated according to the Kaplan-Meier method. Categorical data were compared through the chi-square test, while numerical data were compared through the Kruskal-Wallis H test. All analyses were performed with SPSS^®^ 19.0.

## Results

### Clinical Characteristics

According to the records of West China Hospital, 15 eligible patients were included in our study from 2008 to 2018. The clinical features and the treatment are summarized in Table 1. The median body mass index and the age of 15 patients were 21.3 kg/m^2^ (range, 17.7–30.1 kg/m^2^) and 28 years (range, 13–80 years), respectively. The median age of patients with LCTs was 28 years (range, 13–80 years) while that of patients with SCTs was 27 years (range, 18–50 years). One patient with an unclassified TSCST was diagnosed at 40 years. In our series, we did not find significant differences between age and histopathological differentiation (*p* = 0.901, Kruskal-Wallis H). The left testis was involved in 5 (33.3%) patients, while the right testis was involved in 10 (66.7%) patients. With regard to the clinical presentation, a testis mass was the main symptom in all cases and was accompanied by pain in 2, varicocele in 2, urinary incontinence in 1, testicular hydrocele in 1, gynecomastia in 1, and cryptorchidism in 1. The median diameter of the mass was 1.5 cm (range 0.5–5.0 cm).

**TABLE 1:** Clinical features and treatment in 15 patients with TSCSTs.

Case	Age (y)	BMI (kg/m^2^)	Side	Clinical history	Treatment	Size (cm)	Stage	Histology	Group (No. high-risk features)	Follow-up
1	40	29.0	Right	Testis mass	Orchiectomy	4.0	II	Unclassified	High-risk group (0)	LTF
2	46	24.9	Right	Testis mass Urinary incontinence Bilateral varicocele	Orchiectomy	3.3	I	LCT	Low-risk group (0)	Alive and NED at 97 mo
3	25	Not available	Left	Testis mass Serum testosterone and estradiol levels were abnormal	Orchiectomy	4.1	I	LCT	Low-risk group (0)	LTF
4	18	21.3	Left	Testis mass and pain Serum LDH level was abnormal	Orchiectomy	3.0	I	SCT	Low-risk group (0)	Alive and NED at 88 mo
5	25	17.7	Right	Testis mass	Orchiectomy	1.0	I	SCT	Low-risk group (0)	LTF
6	26	Not available	Left	Testis mass	Orchiectomy	0.5	I	LCT	Low-risk group (0)	LTF
7	80	18.0	Right	Testis mass and pain Serum LDH level was abnormal	Orchiectomy	1.0	I	LCT	Low-risk group (0)	Alive and NED at 80mo
8	69	Not available	Right	Testis mass Left testicular hydrocele	Orchiectomy	3.7	I	LCT	Low-risk group (0)	LTF
9	50	Not available	Right	Testis mass Left varicocele	Orchiectomy	0.5	I	SCT	Low-risk group (0)	Alive and NED at 46 mo
10	50	21.3	Right	Testis mass	Orchiectomy	1.3	II	SCT	High-risk group (3)	Progress at 40 mo
11	65	30.1	Left	Testis mass Gynecomastia	Orchiectomy	1.7	I	LCT	Low-risk group (0)	Alive and NED at 38 mo
12	13	19.1	Left	Testis mass Serum testosterone and estradiol levels were abnormal	Orchiectomy	5.0	I	LCT	Low-risk group (0)	Alive and NED at 37 mo
13	27	20.4	Right	Testis mass	Orchiectomy	1.5	I	SCT	Low-risk group (0)	Alive at 24 mo
14	28	22.0	Right	Testis mass Left cryptorchidism	Orchiectomy	1.0	I	LCT	Low-risk group (0)	Alive at 5 mo
15	19	22.6	Right	Testis mass	Orchiectomy	0.8	I	LCT	Low-risk group (1)	Alive at 13mo

*LDH, lactate dehydrogenase; LCT, Leydig cell tumor; SCT, Sertoli cell tumor; LTF, lost to follow-up; NED, no evidence of disease.*

Tumor markers, such as AFP, β-HCG, and LDH, were normal in all patients before surgery except 2 patients with increased LDH levels. Hormone assays were performed preoperatively in 2 patients, and both showed high preoperative testosterone and estradiol levels. However, the sex hormone levels of patients with gynecomastia were unavailable. All patients received radiological evaluation before the surgical approach, which found suspicious para-aortic lymph node metastases in cases 1 and 10.

### Treatment and Histopathological Findings

Orchiectomy was performed in all patients, and after that, no adjuvant treatment was performed. We recommended RPLND for the two patients (cases 1 and 10) with para-aortic lymph node metastases on radiological evaluation. However, both of them refused to receive RPLND. The pathologic evaluation was performed for all patients after surgery. LCTs were the leading histopathological entities (*n* = 9; 60%), followed by SCTs (*n* = 5; 33%). In one patient (7%), an unclassified TSCST with incomplete differentiation was described. Mitosis (5–8 mitoses per 10 high-power fields) and moderate nuclear atypia were observed in case 10. Tumor necrosis was identified in cases 10 and 15. None of the patients had angiolymphatic invasion or infiltrating margins. Of the 15 patients, 13 had no or 1 high-risk feature and no evidence of retroperitoneal disease on radiological evaluation, and they were classified as the low-risk group. Two patients (cases 1 and 10) were classified into the high-risk group due to 2 or more high-risk features (case 10) or retroperitoneal disease on radiological evaluation (case 1) (Table 1). In all patients, sufficient clinical and pathological data were available for staging according to the National Comprehensive Cancer Network staging system. Thirteen patients (86.7%) were categorized as stage I, and 2 patients (13.3%) with para-aortic lymph node metastases were categorized as stage II.

### Prognosis

**FIGURE 1:**
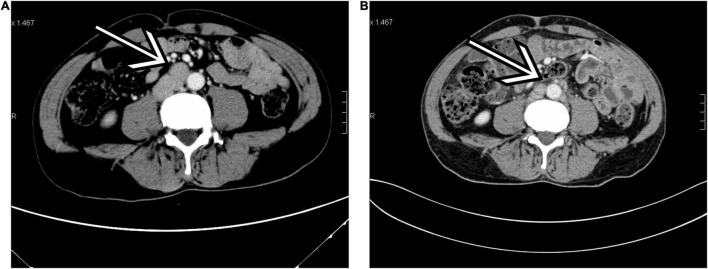
A 50-year-old man with Sertoli cell tumors (SCT; case 10). **(A)** Transverse contrast-enhanced CT (arterial phase) performed before orchiectomy showed enlarged para-aortic lymph nodes (arrow). **(B)** The same level of contrast-enhanced CT (arterial phase) performed 40 months after orchiectomy found that the enlarged para-aortic lymph nodes had diminished (arrow). **p < 0.01, ***p < 0.001.

**FIGURE 2:**
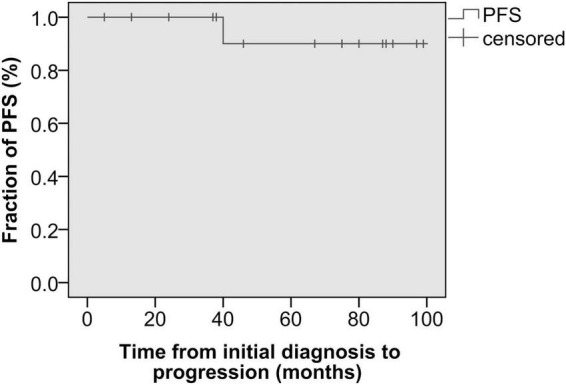
Progression-free survival curve—the time from the initial diagnosis to progression.

Follow-up information was available for 10 patients with a median follow-up of 39.0 months (range, 5–97 months), which showed 10 alive patients. In the low-risk group (*n* = 13), all patients were alive, and no evidence of disease was observed during follow-up, except for 4 patients who were lost to follow-up. In the high-risk group (*n* = 2), one patient (case 10) had a novel inguinal mass with pain at 40 months after orchiectomy and was identified as having progression (inguinal lymph node metastases) through radiological evaluation. Comparing the preoperative and postoperative CT scans at 40 months in this patient (case 10), we found that the enlarged para-aortic lymph nodes detected before orchiectomy had diminished (Figure 1). Another patient (case 1) was alive without clinical or radiographic evidence of progression until a loss to follow-up at 108 months. The 3-r and 5-year PFSs were 100 and 90.0%, respectively (Figure 2). No additional surgery or adjuvant therapy was performed for any patients during follow-up except the one with progression. All patients without follow-up information were due to loss of contact with patients and their families. Table 2 summarizes several studies of TSCSTs published previously.

**TABLE 2:** Summary of TSCSTs previously reported in the literature.

References	Country	No. Cases	Histology	Age (y)	Size (cm)	Treatment	Follow-up (mo)	Disease Status
Harms et al. 1997 (3)	Germany	29	SCTs (*n* = 18) GCTs (*n* = 11)	SCTs: mean, 4.2 (range, 0-14) GCTs: mean, 0.4 (range, 0-3)	SCTs: mean, 1.9 (range, 0.7-4.0) GCTs: mean, 2.0 (range, 1.0-2.2)	Orchiectomy (*n* = 23) Orchiectomy+ RPLND (*n* = 1) Orchiectomy+ RPLND+Ch (*n* = 1) Enucleation (*n* = 2)	SCTs: mean, 39.3 (range, 9-96) GCTs: mean, 46.1 (range, 7-117)	Alive and NED (*n* = 24)
Silberstein et al. 2014 (4)	United States	48	Unclassified (*n* = 5) LCTs (*n* = 28) SCTs (*n* = 13) GCTs (*n* = 2)	Low risk (median, 37) High risk (median, 48)	Low risk (median, 1.5) High risk (median, 5.0)	Orchiectomy (*n* = 37) Orchiectomy+ RPLND (*n* = 11)	Low risk (median, 15) High risk (median, 67)	Alive and NED (*n* = 43) Recurrence (*n* = 2) DOD (*n* = 3)
Kao et al. 2014 (7)	United States	20	SCTs	Mean, 37.0; median, 39 (range, 0.01-12)	Mean,1.7; (range, 0.5-6.0)	Orchiectomy (*n* = 14) Excisional biopsy (*n* = 3) Wedge biopsy (*n* = 2) Orchiectomy+ RPLND (*n* = 1)	Mean, 73.0 (range, 3-192)	Alive and NED (*n* = 9) Alive (*n* = 5) DOD (*n* = 1)
Nason et al. 2017 (8)	Ireland	22	LCTs	Median, 45.0	Not available	Testis-sparing surgery (*n* = 21) Testis-sparing surgery+orchiectomy (*n* = 1)	Median, 102.0 (range, 1-237)	Alive and NED (*n* = 17) Recurrence (*n* = 1) Died with NED (*n* = 4)
Kim et al. 1985 (9)	United States	40	LCTs	Mean, 46.5 (range, 2-90)	Mean, 3.0 (range, 0.5-10.0)	Not available	Mean, 48.0 (range, 2-264)	Alive and NED (*n* = 25)
Young et al. 1998 (10)	United States	60	SCTs	Mean, 45 (range, 15-80)	Mean, 3.6 (range, 0.3-15)	Not available	Mean, 100.8	DOD (*n* = 5) Alive and NED (*n* = 9) Alive (*n* = 4) DOD (*n* = 3)
Carmignani et al. 2006 (11)	Italy	24	LCTs	Mean, 37.8 (range, 22-61)	Mean, 1.5 (range, 0.5-4.5)	Orchiectomy (*n* = 24)	Mean, 117 (range, 11-241)	Alive and NED (*n* = 24)
Featherstone et al. 2009 (12)	United Kingdom	38	LCTs (*n* = 31) SCTs (*n* = 6) LCCSCT (*n* = 1)	Mean, 43 (range, 18-79)	Mean, 1.7 (range, 0.5-8.0)	Orchiectomy (*n* = 36) Surgical enucleation (*n* = 2)	Median, 76.6 (range, 16.8-300)	Alive (*n* = 36)
Cornejo et al. 2014 (13)	United States	32	GCTs	Mean, 40.0 (range, 14-87)	Mean, 2.8 (range, 0.5-6.0)	Orchiectomy (*n* = 30) Wedge excision (*n* = 2)	Mean, 51.0 (range, 1-169)	Alive and NED (*n* = 18) Metastasis (*n* = 1)

*LCTs, Leydig cell tumors; SCTs, Sertoli cell tumors; GCTs, granulosa cell tumors; LCCSCT, large cell calcifying Sertoli cell tumor; RPLND, retroperitoneal lymph nodes dissection; Ch, chemotherapy; NED, no evidence of disease; DOD, died of disease.*

## Discussion

Testicular sex cord-stromal tumors (TSCSTs) comprise approximately 4% of all testicular tumors, while the frequency increases to 8% in children (1). From 2008 to 2018, a total of 877 patients were diagnosed with testicular tumors at West China Hospital, and only 15 of them (1.7%) had TSCST, slightly lower than the overall proportion. A review of 9 studies published previously that included 313 cases of TSCSTs was performed with attention to the clinical features, treatment, and prognostic outcomes (3, 4, 7–13). In general, there is a wide age range for TSCSTs, with ages in two contemporary series ranging between 14 and 87 years (7, 13). LCTs constitute approximately 3% of testicular neoplasms and occur in both adults and children. SCTs account for less than 1% of testicular tumors, and they occur both in children and in middle-aged adults and could be malignant in both (9, 16, 17). In our study, the median ages of patients with LCTs and SCTs were 28 and 27 years, respectively. However, we did not find significant differences between these two groups in age.

The pathogenesis of TSCSTs is poorly understood. Recently, a single recurrent somatic mutation in the *FOXL2* gene was identified in almost all morphologically adult granulosa cell tumors of the ovary (18, 19). *FOXL2* is a forkhead transcription factor that contains a fork-head DNA-binding domain, plays a role in ovarian development and function, and is essential for granulosa cell differentiation (18). Nevertheless, little is known about the biological activity of *FOXL2* in TSCSTs. Kalfa et al. (20) described aberrant and nuclear *FOXL2* expression in three testicular juvenile granulosa cell tumors. In their study, however, whether *FOXL2* alone had the capacity to induce the transformation of testis cells into malignant granulosa cells remained unclear. Moreover, Boyer et al. (21) reported that *Ctnnb1* mutant mice with the loss of *phosphatase and tensin homolog* (*Pten*) expression in their Sertoli cells had developed testicular GCTs. Richards et al. (22) confirmed that except for *Pten* loss, *Kras* activation in Sertoli cells of *Ctnnb1* mutant mice could also induce GCTs. However, despite these genetic findings, the pathogenesis of TSCSTs remains unclear.

Testicular sex cord-stromal tumors generally do not have aggressive behavior. LCTs are less aggressive in children than adults, and approximately 10% of LCTs present with malignant characteristics (23). Kim et al. (9) reported a clinicopathologic analysis of 40 patients with LCTs and found that 5 of them died of metastasis. Additionally, SCTs are considered well-differentiated tumors. However, 4 cases with metastasis were described in two series of patients with SCTs (7, 10). Some authors found that malignant tumors were larger, often had an infiltrative margin and spread beyond the testis, frequently exhibited blood vessel or lymphatic invasion, had a greater degree of cellular atypia and necrosis, and a higher mitotic rate than benign tumors (9, 10, 24). The wide morphological range of TSCSTs makes their diagnosis difficult, and the challenge remains in characterizing patients with benign or malignant tumors.

In most TSCSTs, the clinical presentation is similar to testicular germ cell tumors, of which a mass of testis was the most common symptom (16). Children with LCTs generally present with endocrinologic symptoms (virilization and gynecomastia) due to excessive secretion of sex steroids, while testicular mass and gynecomastia are more common in adult men (9, 11). In view of these findings, preoperative hormone assays may be helpful in the diagnosis of TSCSTs, especially LCTs. However, in our series, only 2 patients received hormone assays, and both showed increased serum estradiol and testosterone levels without any presentation of endocrinologic symptoms. It is unfortunate that the hormone levels of the patient with gynecomastia were unavailable. Moreover, patients with SCTs usually present with a testicular mass and gynecomastia induced by estrogen secretion of the tumor, which were not found in our patients. In men, large-cell calcifying SCTs can be found in patients with Peutz-Jeghers syndrome or the Carney complex (25, 26). However, no clinical data indicated that Peutz-Jeghers syndrome or the Carney complex existed in our patients. Radiological evaluation is helpful in the early diagnosis of TSCSTs. However, there are no pathognomonic radiological features that can accurately determine the presence of a TSCST and differentiate the benign and malignant forms of TSCSTs (8, 27).

The treatment for TSCSTs is still controversial around the world. In principle, orchiectomy constitutes the main therapy for TSCSTs (15). However, Cecchetto et al. (17) suggested that a testis-sparing surgery should be taken into consideration when preoperative normal levels of AFP can rule out a malignant germ cell tumor and ultrasound shows a small and encapsulated mass. Furthermore, Nason et al. (8) found only one local recurrence after the organ-preserving procedure in 22 patients with LCTs, and no progressive disease was reported. Another multicenter retrospective study that includes 204 cases also observed benign behavior and favorable prognosis of LCTs, in which testis-sparing surgery was performed for all patients (28). In addition, Grogg et al. (29) performed a systematic literature review and meta-analysis of outcomes in 435 patients with SCTs. They found that few local recurrences after testis-sparing surgery without adjuvant therapy could be regarded as a standard of care. Age, tumor size, necrosis, tumor extension to the spermatic cord, angiolymphatic invasion, and mitotic index are predictive of metastatic disease. The American Urological Association guidelines have recommended that testis-sparing surgery combined with a frozen-section is an option for preservation of hormonal function and fertility in patients with solitary testis or bilateral synchronous malignancy, especially for patients with masses < 2.0 cm, equivocal ultrasound/physical examination findings and negative tumor markers (β-HCG and AFP) (Grade C) (30). Nevertheless, it is worth noting that this strategy has not been validated prospectively, and few data have demonstrated its long-term oncologic and functional superiorities for TSCST (31). Moreover, the value of RPLND for patients with TSCSTs is uncertain. Silberstein et al. (4) found that patients with one or no high-risk feature can be safely observed without RPLND. Early RPLND may be beneficial in those with two or more high-risk features. However, in another series of 52 patients with LCTs, no retroperitoneal disease was observed after pathologic evaluation in 5 patients who received RPLND (i.e., 2 patients with a retroperitoneal disease on preoperative radiology) (32). In our study, all patients were treated with orchiectomy without any adjuvant therapy after surgery. No patient developed novel metastatic disease or disease-related death during follow-up, except that 1 patient in the high-risk group had inguinal lymph node metastases at 40 months. Interestingly, we found that enlarged para-aortic lymph node metastases had diminished when compared with the preoperative and postoperative CT scans at 40 months in this patient (case 10), although the patient refused to receive RPLND after orchiectomy. Therefore, in our opinion, the value of preoperative radiological evaluation in predicting the malignancy and prognosis of TSCSTs is limited. RPLND should be reserved for patients with stage II TSCC and those with 2 or more high-risk features.

In general, the majority of studies, such as our study, confirmed the benign biological behavior and favorable prognosis of TSCSTs. Kim et al. (9) found that the size, cellular atypia, necrosis, mitosis, and invasion of the tumor are related to the degree of malignancy of TSCSTs, which may predict the prognosis of the tumor. In addition, patients with LCTs or SCTs could present with hormonal manifestations as the endocrine function of tumors (9, 11). Therefore, hormone assays have potential clinical value in predicting the subtype of TSCSTs. However, it is unclear whether serum hormone levels are a prognostic factor. With regard to GCTs, after a systematic review of published case series data, Grogg et al. (33) reported that tumor size, presence of angiolymphatic invasion, or gynecomastia represent risk factors for metastatic disease. In our study, only one patient with preoperative lymph node metastases had progression during follow-up, indicating that lymph node metastases may be a dominant factor for the prognosis of TSCSTs.

We must acknowledge several limitations of our study. First, only 15 patients were included in our study, and prospective studies with larger samples are needed in the future. Second, some patient data, such as serum hormone levels, were not available because of the limitation of retrospective studies. Finally, some patients were lost to follow-up even if we tried to contact their relatives and families, which may reduce the accuracy of our findings.

## Conclusion

Our study confirmed that TSCST at stages I and II is a rare subtype with benign behavior and a favorable prognosis in the Chinese population. However, lymph node metastases may be the dominant risk factor for patients with TSCSTs. Large-scale and multicenter prospective studies are needed to further evaluate the efficacy and safety of orchiectomy, testis-sparing surgery, and adjuvant therapy for TSCST.

## Data Availability Statement

The original contributions presented in the study are included in the article/supplementary material, further inquiries can be directed to the corresponding author/s.

## Ethics Statement

The studies involving human participants were reviewed and approved by the Ethics Committee on Biomedical Research, West China Hospital of Sichuan University. Written informed consent to participate in this study was provided by the participants’ legal guardian/next of kin. Written informed consent was obtained from the individual(s), and minor(s)’ legal guardian/next of kin, for the publication of any potentially identifiable images or data included in this article.

## Author Contributions

YH, BC, and DC: conception and design, data analysis, interpretation, and provision of study materials or patients. QD, LL, and QW: administrative support. YH, BC, DC, ZC, JG, and JL: collection and assembly of data. YH, BC, DC, ZC, JL, JG, QD, LL, and QW: manuscript writing. All authors contributed to the article and approved the submitted version.

## Conflict of Interest

The authors declare that the research was conducted in the absence of any commercial or financial relationships that could be construed as a potential conflict of interest.

## Publisher’s Note

All claims expressed in this article are solely those of the authors and do not necessarily represent those of their affiliated organizations, or those of the publisher, the editors and the reviewers. Any product that may be evaluated in this article, or claim that may be made by its manufacturer, is not guaranteed or endorsed by the publisher.
